# From screen to sweat: how platform features translate into exercise behavior via perceived value in online fitness live streaming

**DOI:** 10.3389/fpsyg.2026.1869785

**Published:** 2026-08-17

**Authors:** LiRu Zhang, Yi Du, Yongxiao Li

**Affiliations:** 1 The Sports Academy, Jiangsu Ocean University, Lianyungang, Jiangsu, China; 2 The Sports Academy, Zhangjiakou University, Zhangjiakou, Hebei, China; 3 School of General Education, Taizhou Vocational College of Science & Technology, Taizhou, Zhejiang, China

**Keywords:** exercise behavior, interactivity, online fitness live streaming, perceived value, Stimulus-Organism-Response framework, university students

## Abstract

Drawing on the Stimulus-Organism-Response (SOR) framework and perceived value theory, this study examined how three online fitness live streaming (OFLS) platform features—content quality, visual and audio effects, and interactivity—relate to exercise behavior among university students. Using a cross-sectional online survey, data were collected from 386 Chinese university students (49.5% male) with prior OFLS experience. Validated measures were translated into Chinese via a forward–backward procedure and assessed on a 5-point Likert scale. Analyses included confirmatory factor analysis, hierarchical regression, and path analysis with bias-corrected bootstrapping (5,000 resamples). Results indicated that all three platform features were positively associated with perceived value (*β* = 0.346, 0.451, and 0.328, respectively; all *ps* < 0.001) and with exercise behavior (*β* = 0.306, 0.168, and 0.256, respectively; all *ps* < 0.001). Perceived value was positively associated with exercise behavior (*β* = 0.299, *p* < 0.001) and partially mediated each feature–behavior relationship. The specific indirect effects were: content quality (*β* = 0.104, 95% CI [0.058, 0.156]), visual and audio effects (*β* = 0.135, 95% CI [0.072, 0.195]), and interactivity (*β* = 0.098, 95% CI [0.047, 0.148]). The full model explained 60.8% of the variance in exercise behavior. These findings suggest that OFLS platform features are linked to exercise behavior through coexisting direct and value-mediated pathways. Content quality and interactivity showed comparatively stronger direct effects, whereas visual and audio effects exhibited the strongest indirect effect via perceived value. Implications for digital health platform design and university wellness programs are discussed.

## 1 Introduction

The rapid advancement of information technology has propelled live streaming into various facets of social life, fundamentally reshaping human lifestyles and interactions (Yu et al., 2025). Amidst a growing public emphasis on health, traditional fitness models have increasingly integrated with digital means, giving rise to the innovative phenomenon of online fitness live streaming (OFLS) (Wang L. et al., 2025). Unlike pre-recorded videos, OFLS transcends spatial and temporal constraints while offering real-time interactivity, fostering an immersive environment that enhances user engagement through immediate feedback and social connectivity (Chen and Lin, 2018). These affordances are particularly relevant for university students, a demographic facing elevated risks of physical inactivity due to academic pressures and restricted access to offline facilities (Zhang et al., 2023). Consequently, investigating how this digital modality relates to exercise outcomes for this population represents a critical public health imperative.

Despite the growing popularity of OFLS, existing literature presents a significant gap. Previous studies have predominantly utilized the Technology Acceptance Model (TAM) to predict users’ initial intention to adopt these platforms based on perceived ease of use and usefulness (Adnan et al., 2025; Al-Adwan et al., 2023; Davis et al., 1989). However, TAM often falls short in explaining the complex, sustained behavioral changes required for effective physical training because it does not fully capture the experiential and psychological trade-offs consumers make during service consumption (Taherdoost, 2018). More critically, the transition from “intending to use a fitness platform” to “actually engaging in physical exercise” remains poorly understood. While prior research has extensively examined users’ adoption intentions, evidence remains scarce on how specific platform design features relate to actual exercise behavior—particularly in the context of real-time interactive streaming, where the distinction between “watching” and “doing” is critical (Elsotouhy et al., 2024).

To bridge this gap, this study posits that understanding the efficacy of OFLS as a digital health intervention requires a theoretical lens that accounts for the dynamic interaction between environmental stimuli and internal cognitive states. Accordingly, we integrate the Stimulus-Organism-Response (SOR) framework with the theory of perceived value to delineate the potential mechanisms driving exercise outcomes (Jacoby, 2002; Mehrabian and Russell, 1974). The SOR framework suggests that external environmental factors (Stimulus) trigger an internal emotional and cognitive processing state (Organism), which subsequently influences the behavioral reaction (Response). In the context of digital health interventions, this framework is well suited for examining how platform affordances are psychologically processed into health behavior change.

In this context, OFLS features—such as content quality, visual and audio effects, and interactivity—act as environmental stimuli. The crucial mediating “Organism” is identified as perceived value, a multidimensional construct reflecting the user’s subjective assessment of the trade-off between benefits received (functional, emotional, and social) and sacrifices made (Sweeney and Soutar, 2001; Zeithaml, 1988). While perceived value has been extensively applied in e-commerce and service marketing, its specific role in mediating the relationship between live streaming platform features and physical exercise outcomes remains underexplored. Specifically, it remains unclear whether different platform features rely differentially on cognitive appraisal processes when influencing exercise behavior.

This study, therefore, aims to address these identified lacunae by constructing a comprehensive model rooted in the SOR framework. Focusing on university students in economically developed regions with high digital literacy, we seek to:

1. Examine the direct relationships between three OFLS platform features (content quality, visual and audio effects, and interactivity) and exercise behavior.
2. Investigate the mediating role of perceived value in linking OFLS stimuli to exercise outcomes.
3. Explore whether content quality, visual and audio effects, and interactivity exhibit differential direct and value-mediated pathways to exercise behavior.

The following sections develop the theoretical rationale and specific hypotheses for these aims.

### 1.1 Research hypotheses

Drawing upon the SOR framework, we conceptualize OFLS features—content quality, visual and audio effects, and interactivity—as environmental stimuli (S) that influence the user’s internal cognitive state, represented by perceived value (O), which subsequently relates to their behavioral response (R), manifested as exercise behavior (Elsotouhy et al., 2024; Mehrabian and Russell, 1974).

*Content quality and exercise behavior.* In the context of digital health, the quality of information provided by instructors serves as a critical antecedent to user engagement. According to the Information Systems Success Model, high-quality content reduces information asymmetry and uncertainty, which is particularly vital in fitness training where professional guidance is required to prevent injury (Delone and McLean, 2003; Zhou et al., 2019). Professionally structured content that explains exercise physiology in an accessible manner and demonstrates training principles through precise corrections can enhance users’ trust and cognitive absorption (Wongkitrungrueng and Assarut, 2020). When users perceive the content as instrumental to achieving their health goals, their intrinsic motivation may be strengthened. Based on this, we propose the following hypothesis:

*H1*: The quality of OFLS content is positively related to exercise behavior.

*Visual and audio effects and exercise behavior.* Beyond informational content, the technical affordances of the OFLS environment, specifically visual and auditory atmospherics, play a pivotal role in shaping user experience. From the perspective of Flow Theory, high-fidelity visual and audio effects contribute to a sense of telepresence, blurring the boundary between the virtual and physical worlds and facilitating a state of “flow” or deep immersion (Ye et al., 2020). High-definition, lag-free visuals allow users to observe movement details clearly, ensuring safety and imitation accuracy, while auditory cues such as rhythmic music and clear vocal instructions regulate users’ arousal levels and focus (Lei et al., 2025). Research suggests that superior visual and audio effects are associated with enhanced sensory enjoyment and reduced disruption of the exercise experience, thereby fostering habit formation (Cai et al., 2022). Based on this, we propose the following hypothesis:

*H2*: The visual and audio effects of OFLS are positively related to exercise behavior.

*Interactivity and exercise behavior.* Interactivity represents a core differentiating feature of OFLS compared to pre-recorded content, establishing a bidirectional communication mechanism that transforms users from passive recipients into active participants (Yin and Xiao, 2025). This active participation directly heightens user immersion and engagement, fostering increased interest and persistence in fitness activities. Furthermore, interactive features such as bullet comments, virtual gifts, and chat enable users to interact with peers and instructors during the stream, cultivating a communal atmosphere that may mitigate feelings of loneliness associated with solitary exercise (Holt et al., 2024; Kaveladze et al., 2022). From a theoretical standpoint, a highly interactive environment may also stimulate users’ self-efficacy, as receiving real-time affirmation and encouragement reinforces their sense of competence (Bandura, 1978). Additionally, interactivity enables greater personalization of fitness content, as instructors can dynamically adjust exercise intensity and motivational strategies based on real-time viewer input (Yin and Xiao, 2025). Based on the critical role of interactivity outlined above, we propose the following hypothesis:

*H3*: The interactivity of OFLS is positively related to exercise behavior.

The mediating role of perceived value. The preceding hypotheses posit direct associations between each platform feature and exercise behavior. However, these relationships may also operate through cognitive appraisal mechanisms. Unlike general OFLS content consumption, user engagement and the development of exercise habits are likely influenced not only by platform technical features but also by the subjective value users derive from the experience (Mrvelj et al., 2020; Zhou and Amirpour, 2025). Prior research in live streaming contexts indicates that high-quality content, smooth audiovisual delivery, and interactive features each contribute to users’ overall perceived value (Chen et al., 2024; Su et al., 2026; Tian and Frank, 2024).

Perceived value is defined as a user’s subjective assessment after weighing costs and benefits (Jensen, 2001; Ulaga and Chacour, 2001). Within the SOR framework, it functions as a cognitive-evaluative mechanism that connects environmental stimuli to behavioral responses. Users comprehensively evaluate their experience derived from content, audiovisuals, and interaction, and this evaluation subsequently informs their behavioral choices (Kundu and Datta, 2015; Te, 2022). When users perceive high value from a live streaming platform, they may increase their exercise frequency and intensity. Thus, perceived value is theorized as a key mediator through which OFLS platform features relate to exercise behavior. Accordingly, we propose the following hypotheses:

*H4*: Perceived value mediates the relationship between OFLS platform features and exercise behavior.

*H4a*: Perceived value mediates the relationship between OFLS content quality and exercise behavior.

*H4b*: Perceived value mediates the relationship between OFLS visual and audio effects and exercise behavior.

*H4c*: Perceived value mediates the relationship between OFLS interactivity and exercise behavior.

**Figure 1:**
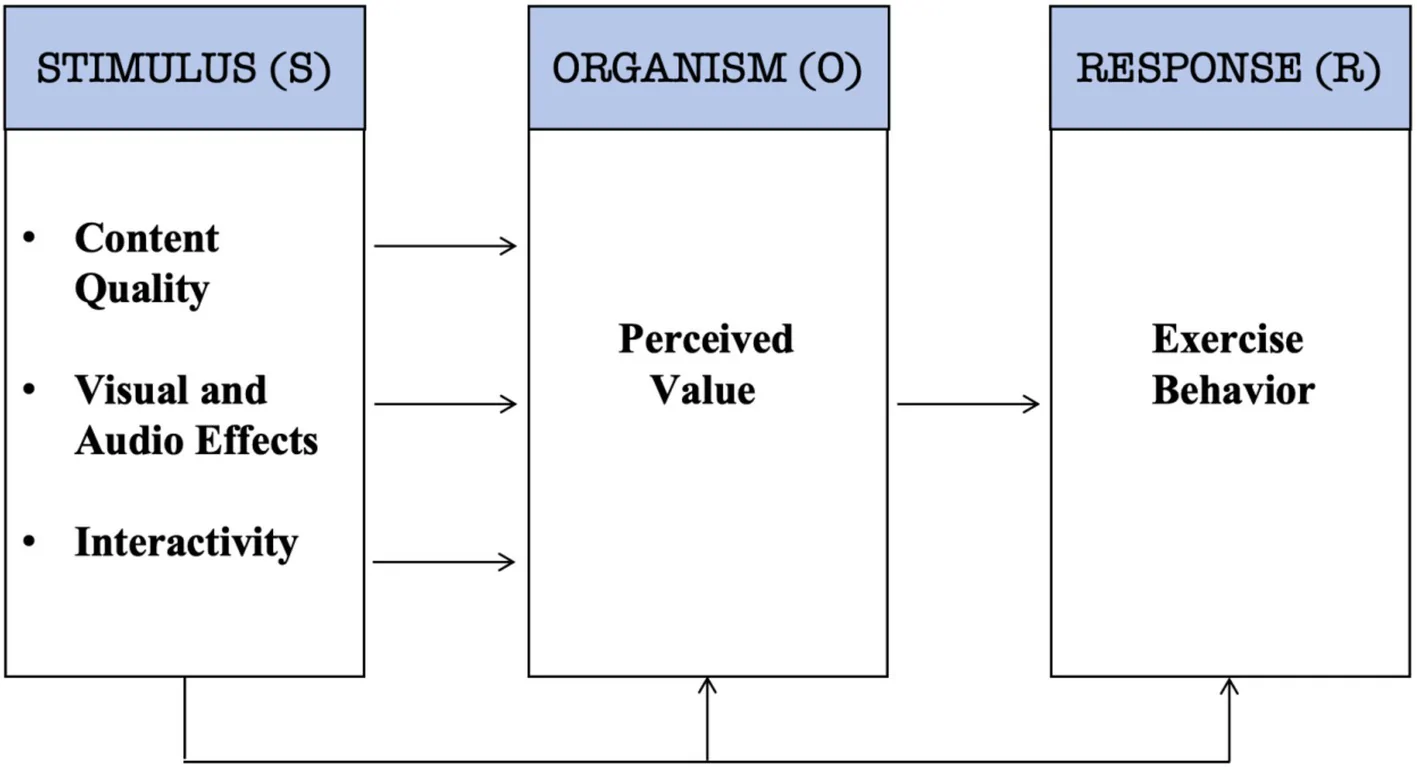
Theoretical model.

The theoretical model of this study is illustrated in Figure 1.

In summary, this study examines a conceptual model in which three OFLS platform features (content quality, visual and audio effects, and interactivity) serve as exogenous stimuli that are hypothesized to predict exercise behavior both directly and indirectly through perceived value as a mediator. The Methods section below describes the procedures used to test these hypotheses.

## 2 Methods

### 2.1 Study design and participants

This study employed a cross-sectional online survey design. Data were collected between December 2025 and March 2026 using convenience sampling at three universities in China (Jiangsu, Zhejiang, and HeBei). Participants were recruited through a combination of classroom announcements, university social media platforms and direct invitations from student ambassadors. The online questionnaire was administered via Wenjuanxing, a widely used Chinese survey platform.

Inclusion criteria were: (1) enrolled as a full-time undergraduate and junior college student; (2) at least one prior experience of using online fitness live streaming (OFLS) platforms; and (3) voluntary participation with signed informed consent. No monetary incentives were provided; participation was entirely voluntary. Students who had never used OFLS were excluded because the study required prior familiarity with the platform features under investigation.

A total of 410 online questionnaires were distributed, with 394 responses received, yielding a response rate of 96%. This high response rate is attributable to classroom-based recruitment in required general education courses, where instructors allocated 10–15 min of class time for voluntary completion. After excluding 8 invalid questionnaires (3 with excessive missing data, 5 with uniform responses across all items), 386 valid questionnaires were collected. The final sample comprised students from three universities across three provinces. The demographic characteristics are presented in Table 1.

**Table 1:** Descriptive statistics.

Item	Category	Count	Percentage (%)
Gender	Male	191	49.48%
	Female	195	50.52%
Grade level	First-year student	125	32.38%
	Second-year student	96	24.87%
	Third-year student	85	22.02%
	Final-year student	80	20.73%
Weekly exercise frequency	0–1 time	94	24.35%
	2–3 times	145	37.56%
	4–5 times	98	25.39%
	Over 5 times	49	12.69%
Fitness goal	Body sculpting	212	54.92%
	Muscle gain	97	25.13%
	Relaxation	77	19.95%
Weekly live stream viewing frequency	1–2 times	80	20.73%
	3–4 times	210	54.40%
	Over 5 times	96	24.87%
Preferred live stream type	Body sculpting	161	41.71%
	Muscle gain	161	41.71%
	Yoga/relaxation	64	16.58%

All participants were informed about the study procedures, potential risks, and study objectives. They provided signed informed consent prior to participation. The study protocol was approved by the institutional review board of Jiangsu Ocean University (protocol number: 20251210) and adhered to the ethical principles of the Declaration of Helsinki.

### 2.2 Measures

#### 2.2.1 Instrument development and translation

The questionnaire was structured into three sections: (1) basic demographic information (gender, age, grade level, major, and frequency of fitness exercise); (2) OFLS platform feature evaluations; and (3) exercise behavior. All construct items were adapted from validated English-language scales (see Tables 2–4 for sources). Because no authorized Chinese versions were available for the specific item sets used, we followed a standard forward–backward translation procedure (Brislin, 1970). Two bilingual researchers independently translated the items into Chinese; discrepancies were resolved through discussion. A third bilingual researcher then back-translated the Chinese items into English to verify semantic equivalence. The translated questionnaire was pilot-tested with 30 college students to assess item clarity and comprehension; minor wording adjustments were made based on feedback. All construct items were rated on a five-point Likert scale ranging from 1 (strongly disagree) to 5 (strongly agree).

**Table 2:** Measurement items for stimulus variables.

Variable	Item	Reference source
Live content quality	The fitness instruction content in the live stream is practical	López-Carril et al. (2025)
	The instructor’s explanations are professional and easy to understand	
	The exercise movements are standardized and effectively prevent sports injuries	
Visual and audio effects	The live stream picture is clear and stable, without lagging	Ahumada-Newhart et al. (2024)
	The audio quality is good, and the instructor’s voice is clear and easy to understand	
	The overall visual presentation of the live stream feels comfortable and engaging	
Interactivity	I can interact with the instructor via bullet comments or comments during the live stream	Wang M. et al. (2025)
	The instructor responds to questions or suggestions raised by the audience	
	The live streaming platform provides interactive features like liking and tipping, enhancing participation	

**Table 3:** Measurement items for organism variables.

Variable	Item	Reference source
Perceived value	Online fitness live streaming helps me achieve my fitness goals	Guo and Li (2022)
	Compared to offline fitness, online fitness live streaming is more cost-effective	
	I believe online fitness live streaming is worth using long-term	

**Table 4:** Measurement items for response variables.

Variable	Item	Reference source
Exercise behavior	Since watching online fitness live streams, I have increased my exercise frequency	Rodrigues Sampaio et al. (2020)
	Watching online fitness live streams makes it easier for me to stick to my fitness plan	
	I am more inclined to engage in fitness exercise through online live streaming	

It should be noted that exercise behavior was measured using self-report items only. The absence of objective physical activity indicators constitutes a methodological limitation that is addressed in the Discussion.

1. External Stimulus (S)
2. Organism (O)
3. Response (R)

### 2.3 Analytical strategy

All analyses were conducted using the full sample of 386 valid responses. Statistical analyses were performed using jamovi (version 2.7.33) and IBM SPSS Statistics (version 26).

#### 2.3.1 Measurement model assessment

Construct validity was examined through confirmatory factor analysis (CFA), with standardized factor loadings > 0.70 and average variance extracted (AVE) > 0.50 indicating adequate convergence. Discriminant validity was evaluated using the Fornell–Larcker criterion (Fornell and Larcker, 1981), supplemented by the heterotrait-monotrait ratio of correlations (Henseler et al., 2015), with HTMT < 0.85 considered supportive of discriminant validity. Model fit was assessed for the overall measurement model—which specified one latent factor for each of the five subscales (live content quality, visual and audio effects, interactivity, perceived value, and exercise behavior)—using *χ*^2^/df, GFI, CFI, TLI, IFI, and RMSEA.

#### 2.3.2 Descriptive and correlational analyses

Frequencies and percentages were computed to describe the demographic characteristics of the participants. Pearson correlation coefficients were computed to examine the bivariate relationships among the key variables. Significance levels were set at *p* < 0.05, *p* < 0.01, and *p* < 0.001.

#### 2.3.3 Regression analyses

Following the recommendations for testing mediation (Frazier et al., 2004), we conducted a three-step hierarchical regression analysis. All analyses in this section were performed on composite scale scores (i.e., the mean of items for each construct). It is important to note that because composite scores retain measurement error, the standardized coefficients reported in Table 5 should be interpreted as preliminary approximations. The definitive structural path coefficients—free from attenuation due to unreliability—were obtained through latent-variable path analysis (Section 2.3.4). In Step 1 (Model 1), perceived value was regressed on the three platform features to establish that the stimuli influence the proposed mediator. In Step 2 (Model 2), exercise behavior was regressed on perceived value to confirm that the mediator predicts the outcome. In Step 3 (Model 3), exercise behavior was regressed simultaneously on all three platform features and perceived value; comparing the beta coefficients of the platform features in Model 3 with their simple direct effects provides a basis for determining whether perceived value partially or completely mediates the relationships. Because the three platform features exhibited moderate intercorrelations, we inspected variance inflation factors (VIFs); values between 1 and 10 are generally considered acceptable, with values exceeding 3 warranting caution regarding multicollinearity (Kline, 2011). All VIF values were below 3.0, indicating no serious multicollinearity.

**Table 5:** Regression analysis results.

Model	Predictor	*β*	SE	*t*	*p*	*R* ^2^	Adjusted *R*^2^
Model 1 (DV: Perceived value)						0.577	0.574
	(Constant)	0.002	0.118	0.016	0.987		
	Live content quality	0.407	0.038	10.615	<0.001		
	Visual and audio effects	0.289	0.036	8.059	<0.001		
	Interactivity	0.337	0.036	9.251	<0.001		
Model 2 (DV: Exercise behavior)						0.504	0.503
	(Constant)	0.819	0.096	8.507	<0.001		
	Perceived value	0.681	0.034	19.759	<0.001		
Model 3 (DV: Exercise behavior)						0.608	0.604
	(Constant)	0.071	0.114	0.616	0.538		
	Live content quality	0.303	0.042	7.291	<0.001		
	Visual and audio effects	0.164	0.041	3.966	<0.001		
	Interactivity	0.244	0.039	6.250	<0.001		
	Perceived value	0.285	0.052	5.469	<0.001		

*β*, standardized regression coefficient; SE, standard error; *R*^2^, coefficient of determination. Model 3 demonstrates partial mediation because all platform features remained significant after perceived value was entered.

#### 2.3.4 Path analysis and mediation testing

Because the three platform features are intercorrelated, testing each mediation pathway separately could yield biased estimates. Therefore, we adopted path analysis as an integrated approach that simultaneously estimates all direct and indirect paths while accounting for the covariances among predictors. Path analysis was conducted using jamovi. Mediation effects were estimated using bias-corrected bootstrapping with 5,000 resamples and 95% confidence intervals; an indirect effect was deemed statistically significant if its confidence interval excluded zero. All statistical tests employed a significance level of *α* = 0.05.

## 3 Results

### 3.1 Descriptive analysis

The study collected 386 valid questionnaires from college students, with a balanced gender distribution (49.48% male, 50.52% female). In terms of grade level, 32.38% of the students came from the first year of university, which was the largest proportion, followed by second-year students (24.87%), third-year students (22.02%), and final-year students (20.73%). The education level variable was omitted because its descriptive content substantially overlapped with grade level. In terms of exercise habits, the majority (37.56%) exercised 2–3 times per week, while 24.35% exercised 0–1 time, 25.39% exercised 4–5 times, and 12.69% exercised over 5 times. Body sculpting was the most common fitness goal (54.92%), with muscle gain (25.13%) and relaxation (19.95%) also significant. The most popular live fitness stream type was body sculpting (41.71%), followed by muscle gain (41.71%) and yoga/relaxation (16.58%). Regarding live stream viewing habits, over half (54.40%) watched 3–4 times per week, 20.73% watched 1–2 times, and 24.87% watched over 5 times. The demographic characteristics of the study are shown in Table 1.

### 3.2 Reliability and validity analysis

Table 6 presents the reliability and validity assessment of the measurement model. To evaluate internal consistency, Cronbach’s *α* was calculated for each construct. The results indicate that *α* values ranged from 0.924 to 0.935, which exceeds the conventional threshold of 0.70 and falls within the “excellent” range (≥0.90), indicating strong measurement reliability. Furthermore, construct validity was examined through Confirmatory Factor Analysis (CFA). As illustrated, the standardized factor loadings for all items fell within the range of 0.875 to 0.921 and were statistically significant. The Composite Reliability (CR) for the latent variables spanned from 0.925 to 0.936, well above the recommended cutoff of 0.70. Additionally, the Average Variance Extracted (AVE) values ranged between 0.804 and 0.829, consistently exceeding the critical benchmark of 0.50. Collectively, these metrics demonstrate that the constructs possess strong construct validity.

**Table 6:** Reliability analysis results for each variable.

Dimension	Item	CFA loadings	Cronbach’s *α*	AVE	CR
Live content quality	A1	0.875	0.924	0.804	0.925
	A2	0.914			
	A3	0.901			
Visual and audio effects	B1	0.916	0.931	0.819	0.931
	B2	0.888			
	B3	0.911			
Interactivity	C1	0.917	0.932	0.821	0.932
	C2	0.885			
	C3	0.916			
Perceived value	D1	0.906	0.934	0.825	0.934
	D2	0.898			
	D3	0.921			
Exercise behavior	E1	0.915	0.935	0.829	0.936
	E2	0.900			
	E3	0.916			

Table 7 presents the assessment of discriminant validity based on the Fornell–Larcker criterion. The diagonal elements display the square roots of the Average Variance Extracted for each construct, ranging from a minimum of 0.897 for Live Content Quality to a maximum of 0.910 for exercise behavior. According to this criterion, the diagonal value for a specific variable must exceed the correlation coefficients between that variable and all other constructs in the model. As the data indicates, the square root of the AVE for every construct is consistently and substantially higher than its corresponding off-diagonal inter-construct correlations. For instance, the square root of the AVE for exercise behavior is 0.910, which comfortably surpasses its strongest correlation of 0.710 with perceived value. Additionally, because the Fornell–Larcker criterion has been criticized for its insensitivity when indicator loadings are high, we supplemented this assessment with the Heterotrait-Monotrait ratio of correlations (Henseler et al., 2015). As shown in Table 8, all HTMT values were well below the conservative threshold of 0.85, further confirming adequate discriminant validity.

**Table 7:** Fornell–Larcker criterion results.

Variable	Live content quality	Visual and audio effects	Interactivity	Perceived value	Exercise behavior
Live content quality	0.897				
Visual and audio effects	0.331	0.905			
Interactivity	0.305	0.366	0.906		
Perceived value	0.568	0.656	0.573	0.908	
Exercise behavior	0.584	0.548	0.563	0.710	0.910

**Table 8:** Heterotrait-Monotrait ratio (HTMT).

Variable	Live content quality	Visual and audio effects	Interactivity	Perceived value	Exercise behavior
Live content quality					
Visual and audio effects	0.356				
Interactivity	0.327	0.393			
Perceived value	0.611	0.704	0.614		
Exercise behavior	0.628	0.587	0.604	0.760	

Table 9 details the goodness-of-fit statistics for the overall measurement model, which specified one latent factor for each subscale (live content quality, visual and audio effects, interactivity, perceived value, and exercise behavior). The ratio of Chi-square to degrees of freedom was calculated at 1.250, which falls well within the ideal range of 1 to 3. Regarding absolute and incremental fit indices, the Goodness of Fit Index yielded a value of 0.967 while the Comparative Fit Index reached 0.996. Similarly, the Tucker-Lewis Index and the Incremental Fit Index were recorded at 0.995 and 0.996, respectively. CFI and TLI values exceeding 0.95 indicate good model fit (Hu and Bentler, 1999). The Root Mean Square Error of Approximation was 0.025, satisfying the stringent criterion of being less than 0.08. These metrics collectively indicate that the proposed model exhibits good fit with the empirical data.

**Table 9:** Construct validity analysis.

Indicator	Reference standard	Model output value	Comparison result
*χ*^2^/df	(1,3)	1.250	Acceptable fit
GFI	>0.90	0.967	Acceptable fit
CFI	>0.90	0.996	Acceptable to good fit
TLI	>0.90	0.995	Acceptable to good fit
IFI	>0.90	0.996	Acceptable to good fit
RMSEA	<0.08	0.025	Acceptable fit

Table 10 outlines the descriptive statistics and normality test results for the five dimensions and their respective measurement items. The analysis reveals that the mean values for the items range from 2.34 to 2.71 with standard deviations extending from 0.924 to 1.044, indicating a relatively consistent dispersion of responses. In terms of normality assessment, the skewness values fluctuate between −0.075 and 0.429, while the kurtosis indices range between −0.522 and 0.067. All absolute values for skewness and kurtosis remain strictly below the rigorous thresholds of 3 and 10, respectively, as suggested by established statistical guidelines (Kline, 2011). These findings collectively demonstrate that the data follows a univariate normal distribution and possesses the requisite distributional characteristics to support further statistical hypothesis testing.

**Table 10:** Descriptive statistics and normality test results for each dimension and measurement item.

Dimension	Item	*M*	SD	Skewness	Kurtosis
Live content quality	A1	2.34	0.924	0.429	0.067
	A2	2.44	0.941	0.389	−0.075
	A3	2.52	0.981	0.330	−0.273
Visual and audio effects	B1	2.66	1.031	0.293	−0.399
	B2	2.71	1.042	0.276	−0.522
	B3	2.41	1.019	0.337	−0.371
Interactivity	C1	2.54	1.001	0.268	−0.418
	C2	2.42	1.006	0.321	−0.436
	C3	2.69	1.009	0.199	−0.394
Perceived value	D1	2.62	1.035	0.222	−0.432
	D2	2.58	1.044	0.322	−0.345
	D3	2.63	1.044	0.230	−0.502
Exercise behavior	E1	2.68	1.02	0.203	−0.489
	E2	2.49	1.002	0.294	−0.488
	E3	2.63	0.973	0.260	−0.185

### 3.3 Correlation analysis

Table 11 presents the Pearson correlation coefficients among the key study variables. All correlations were positive and statistically significant (*p* < 0.001). The correlations among the three platform affordances ranged from 0.305 to 0.366, which are substantially lower than those reported in the initial analysis and alleviate concerns about multicollinearity. The correlation between perceived value and exercise behavior (*r* = 0.710) remained moderately high, which may reflect conceptual overlap between value assessments and behavioral self-reports, as well as potential common method variance. Overall, these correlations indicate meaningful but distinguishable associations among constructs; however, they provide preliminary evidence only and should not be interpreted as confirming directional hypotheses.

**Table 11:** Correlations among variables.

Variable	Live content quality	Visual and audio effects	Interactivity	Perceived value	Exercise behavior
Live content quality	1				
Visual and audio effects	0.331^***^	1			
Interactivity	0.305^***^	0.366^***^	1		
Perceived value	0.568^***^	0.656^***^	0.573^***^	1	
Exercise behavior	0.584^***^	0.548^***^	0.563^***^	0.710^***^	1

^***^p < 0.001.

### 3.4 Regression analysis

The regression results are presented in Table 5. In Model 1, live content quality (*β* = 0.407, *p* < 0.001), visual and audio effects (*β* = 0.289, *p* < 0.001), and interactivity (*β* = 0.337, *p* < 0.001) all positively predicted perceived value, collectively accounting for 57.7% of the variance (*R*^2^ = 0.577). In Model 2, perceived value was a significant positive predictor of exercise behavior (*β* = 0.681, *p* < 0.001), explaining 50.4% of the variance. Critically, Model 3 revealed that when perceived value was included alongside the three platform features, all predictors remained statistically significant: live content quality (*β* = 0.303, *p* < 0.001), visual and audio effects (*β* = 0.164, *p* < 0.001), interactivity (*β* = 0.244, *p* < 0.001), and perceived value (*β* = 0.285, *p* < 0.001). The fact that the platform features remained significant predictors after controlling for perceived value indicates that perceived value partially mediates—rather than fully mediates—the relationships between platform features and exercise behavior. The full model accounted for 60.8% of the variance in exercise behavior (*R*^2^ = 0.608).

**Figure 2:**
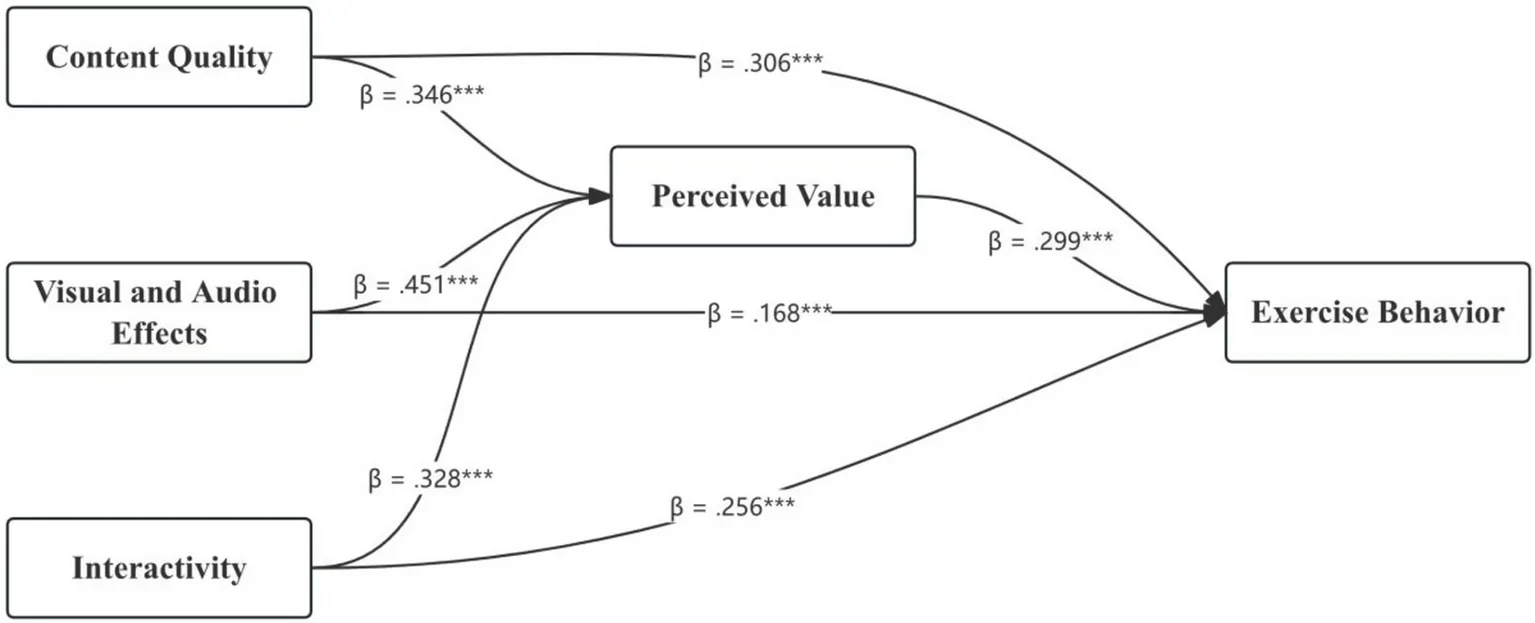
Path analysis results. Standardized coefficients are reported. ^***^p < 0.001.

It should be noted that the standardized coefficients in Model 1 of Table 5 are based on composite scores and therefore differ from the latent-variable estimates reported in the path analysis (Figure 2). Specifically, the latent-variable approach accounts for measurement error in the indicators, which typically produces more accurate—and sometimes larger—structural coefficients. The direct effects reported in Model 3 (Table 5) and Figure 2 are highly congruent because both models simultaneously control for the other predictors when estimating each path. Thus, Table 5 provides preliminary evidence of mediation using observed variables, whereas Figure 2 presents the definitive test using latent variables.

### 3.5 Mediation analysis

Because the three platform features exhibited moderate intercorrelations (Table 11), we adopted path analysis as an integrated approach that simultaneously models all direct and indirect pathways while accounting for the covariances among predictors. Path analysis was conducted using jamovi (version 2.7.33). Mediation effects were estimated using bias-corrected bootstrapping with 5,000 resamples.

Figure 2 presents the standardized parameter estimates for the full path model. All three platform features were significantly associated with perceived value: live content quality (*β* = 0.346, *p* < 0.001), visual and audio effects (*β* = 0.451, p < 0.001), and interactivity (*β* = 0.328, *p* < 0.001). perceived value, in turn, was significantly associated with exercise behavior (*β* = 0.299, *p* < 0.001). Direct effects of the platform features on Exercise Behavior were also significant: live content quality (*β* = 0.306, *p* < 0.001), visual and audio effects (*β* = 0.168, *p* < 0.001), and interactivity (*β* = 0.256, *p* < 0.001).

The indirect (mediated) effects are reported in Table 12. Perceived value significantly mediated the associations between each platform feature and exercise behavior. The specific indirect effects were: live content quality → Perceived value → Exercise behavior (*β* = 0.104, 95% CI [0.058, 0.156], *p* < 0.001); visual and audio effects → Perceived value → Exercise behavior (*β* = 0.135, 95% CI [0.072, 0.195], *p* < 0.001); and interactivity → Perceived value → Exercise behavior (*β* = 0.098, 95% CI [0.047, 0.148], *p* < 0.001). Because the confidence intervals excluded zero for all three indirect effects, partial mediation is supported for each pathway. The proportion of the total effect mediated by perceived value was approximately descriptive: highest for visual and audio effects, followed by interactivity and live content quality. We refrained from formally comparing these proportions because differences in indirect effect sizes across correlated predictors are difficult to interpret and may be influenced by the shared variance among platform features.

**Table 12:** Path analysis: indirect (mediated) effects.

Path	Estimate	SE	95% confidence intervals	*β*	*p*
Live content quality → Perceived value → Exercise behavior	0.107	0.025	[0.058, 0.156]	0.104	<0.001
Visual and audio effects → Perceived value → Exercise behavior	0.134	0.031	[0.072, 0.195]	0.135	<0.001
Interactivity → Perceived value → Exercise behavior	0.097	0.026	[0.047, 0.148]	0.098	<0.001

Confidence intervals are bias-corrected bootstrap intervals based on 5,000 resamples. All indirect effects are significant at *p* < 0.001 because zero falls outside the 95% confidence interval.

## 4 Discussion

This study draws on the Stimulus-Organism-Response (SOR) framework and perceived value theory to examine how platform features in Online Fitness Live Streaming (OFLS) relate to exercise behavior among university students. The findings indicate that content quality, visual and audio effects, and interactivity are each positively associated with both perceived value and exercise behavior, and that perceived value partially mediates these associations. These patterns are broadly consistent with the SOR proposition that environmental stimuli may influence behavioral responses through internal cognitive-evaluative states, though the cross-sectional nature of the data precludes causal conclusions.

In the path model, visual and audio effects demonstrated the strongest association with perceived value, followed by content quality and interactivity. Perceived value, in turn, was positively associated with exercise behavior. Among the direct pathways, content quality showed the strongest direct association with exercise behavior, followed by interactivity and visual and audio effects. The indirect effects through perceived value were statistically significant for all three features, with visual and audio effects showing the strongest indirect pathway, followed by content quality and interactivity. The coexistence of significant direct and indirect pathways across all three features points to a nuanced process in which platform affordances relate to exercise behavior through both value-mediated and more immediate routes.

Content quality exhibited both a substantial direct effect and a significant indirect effect through perceived value. This pattern suggests that professional, well-structured instructional content may be cognitively appraised by users for its instrumental utility before being translated into sustained exercise behavior. Such an interpretation aligns with the Information Systems Success Model, which emphasizes that high-quality information reduces uncertainty and fosters trust in contexts where professional guidance is essential (Delone and McLean, 2003; Zhou et al., 2019). The finding that content quality retained the strongest direct effect on exercise behavior even after accounting for perceived value further underscores its foundational role in digital fitness engagement.

Visual and audio effects occupied a distinctive position in the model: although their direct effect on exercise behavior was comparatively modest, they showed the strongest indirect pathway through perceived value. This configuration suggests that the behavioral relevance of high-quality sensory experiences may operate largely through users’ cognitive evaluation of the service’s professionalism and immersive potential. From the perspective of Flow Theory, superior audiovisual fidelity may facilitate telepresence by blurring the boundary between virtual and physical exercise environments, thereby sustaining user attention and reducing the psychological distance of home-based workouts (Ye et al., 2020). The present data are consistent with the possibility that users derive heightened perceived value from seamless sensory delivery, which in turn supports exercise engagement.

Interactivity displayed a markedly different configuration: its direct effect on exercise behavior was second only to content quality, while its indirect effect through perceived value was the smallest among the three features. This pattern is consistent with theoretical arguments that real-time bidirectional communication in live streaming transforms users from passive viewers into active participants (Yin and Xiao, 2025), potentially activating behavioral engagement through more immediate motivational channels. Social Presence Theory posits that interactive cues—such as coach acknowledgment, bullet-screen comments, and peer recognition—foster a sense of “being together” that sustains participation (Miao and Ma, 2022; Sung and Mayer, 2012). Additionally, the timely validation of effort in interactive settings may reinforce users’ sense of competence, a mechanism conceptually linked to Self-Efficacy Theory (Bandura, 1978). The present findings suggest that interactivity may relate to exercise behavior through pathways that are at least partially independent of deliberate value calculation.

The mediating role of perceived value extends existing e-commerce and social commerce research into the domain of health behavior change (Guo and Li, 2022). In the OFLS context, perceived value appears to function as a cognitive filter that translates platform stimuli into behavioral relevance, echoing the conceptualization of value as a trade-off between benefits received and sacrifices made (Zeithaml, 1988). Because the present study measured perceived value as an aggregate construct, the relative contributions of its functional, emotional, and social sub-dimensions remain to be disentangled. Nevertheless, the consistent significance of indirect effects across all three platform features suggests that value appraisal is a non-trivial mechanism in the exercise behavior formation process.

The present findings may complement Technology Acceptance Model (TAM)-based research by shifting attention from adoption intentions to sustained engagement behaviors. Whereas TAM has been widely used to explain why users initially adopt digital health platforms (Adnan et al., 2025; Al-Adwan et al., 2023), the SOR framework adopted here offers a lens for understanding how specific platform affordances relate to actual exercise behavior once users are immersed in the service. This distinction is particularly relevant for live streaming, where the boundary between “watching” and “doing” is critical (Elsotouhy et al., 2024). The coexistence of direct and mediated pathways observed in this study suggests that post-adoption engagement may be driven by experiential mechanisms that extend beyond the perceived usefulness and ease of use central to TAM.

The varying relative magnitudes of direct and indirect effects across the three platform features raise the possibility of coexisting psychological pathways. Content quality, with its strongest direct effect and comparatively low mediation proportion, may rely more heavily on immediate behavioral activation, whereas interactivity, with its moderate direct effect and intermediate mediation proportion, may engage users through both immediate and value-mediated channels. Visual and audio effects, with the highest mediation proportion, appear to rely more heavily on deliberate cognitive appraisal, contributing to both sensory immersion and subsequent value attribution. Such a pattern is conceptually compatible with dual-process perspectives in which some environmental cues prompt reflective evaluation while others trigger more automatic engagement responses.

From a practical standpoint, the observed association patterns offer several tentative directions for platform design and digital health intervention development. Given that content quality retained the strongest direct effect on exercise behavior and a significant indirect effect through perceived value, platforms may benefit from investing in transparent quality signals—such as instructor certification, structured curriculum design, and progress-tracking interfaces—that make instructional value salient to users. Such features may help users cognitively internalize the utility of the training content, thereby supporting both initial engagement and sustained adherence. The comparatively strong direct effect of interactivity suggests that real-time feedback mechanisms—such as coach acknowledgment by name, peer recognition systems, and dynamic difficulty adjustments responsive to audience input—may represent high-leverage features for behavioral engagement. The present data suggest that these interactive elements are associated with exercise behavior even after controlling for perceived value, implying that their motivational impact may not be fully captured by cognitive value assessment alone. For visual and audio effects, the dominant indirect pathway through perceived value indicates that investment in high-definition, lag-free streaming and immersive production quality may enhance user engagement primarily by elevating the perceived professionalism and trustworthiness of the service. This points to a dual function: technical stability serves as a functional prerequisite for safe movement imitation, while production value contributes to the hedonic quality of the exercise experience. For universities and public health initiatives, OFLS appears to represent a scalable channel for reaching student populations at risk of physical inactivity. The present findings suggest that platform selection and partnership decisions might usefully consider the feature profiles identified here, particularly the behavioral relevance of interactive capabilities.

Several methodological considerations qualify the interpretation of these findings. First, the cross-sectional design precludes causal inference and does not establish temporal precedence. It is plausible that students with pre-existing exercise habits evaluate OFLS features more favorably, meaning that the observed associations may at least partially reflect reverse causality. Second, the reliance on self-reported exercise behavior introduces the possibility of social desirability bias and recall inaccuracy; the absence of objective physical activity indicators constitutes a substantive limitation. Third, the sample was recruited via convenience sampling from three universities in economically developed regions of China, which may restrict generalizability to broader populations with differing digital literacy or fitness culture orientations. Fourth, perceived value was operationalized as an aggregate three-item measure, precluding examination of whether its functional, emotional, and social dimensions play differentiated roles across platform features. Fifth, the three platform features were modeled as separate predictors, yet they likely interact in practice; experimental designs manipulating single features while holding others constant would strengthen causal claims. Future research employing longitudinal, experimental, and multi-method designs is needed to establish temporal precedence, test causal mechanisms, and determine whether these patterns generalize across diverse populations and technological contexts.

## 5 Conclusion

This study examined the associations between Online Fitness Live Streaming platform features and exercise behavior among Chinese university students through the lens of the Stimulus-Organism-Response framework. Based on cross-sectional survey data from 386 participants, the findings suggest that content quality, visual and audio effects, and interactivity are each positively associated with exercise behavior through coexisting direct and perceived-value-mediated pathways.

Path analysis revealed that all three platform features were significantly associated with perceived value (content quality: *β* = 0.346; visual and audio effects: *β* = 0.451; interactivity: *β* = 0.328; all *p* < 0.001), which in turn was positively associated with exercise behavior (*β* = 0.299, *p* < 0.001). Direct effects on exercise behavior were also observed for content quality (*β* = 0.306), interactivity (*β* = 0.256), and visual and audio effects (*β* = 0.168; all *p* < 0.001). Indirect effects through perceived value were significant for all three features, with visual and audio effects showing the largest indirect effect (*β* = 0.135, 95% CI [0.072, 0.195]), followed by content quality (*β* = 0.104, 95% CI [0.058, 0.156]) and interactivity (*β* = 0.098, 95% CI [0.047, 0.148]).

These patterns are consistent with a process in which platform features relate to exercise behavior through multiple psychological pathways, though the cross-sectional design precludes causal interpretation. The varying configurations of direct and indirect effects across features suggest that content quality may operate partly through cognitive value appraisal, interactivity may engage more immediate behavioral mechanisms, and visual and audio effects may function through both sensory immersion and value attribution. Future research employing longitudinal, experimental, and multi-method designs is needed to establish temporal precedence, test causal mechanisms, and determine whether these patterns generalize across diverse populations and technological contexts.

## Data Availability

The data that support the findings of this study are available from the corresponding author upon reasonable request. Requests to access these datasets should be directed to YL, Liyongxiao@jou.edu.cn.

## References

[ref1] Adnan A. Irvine R. E. Williams A. Harris M. Antonacci G. (2025). Improving acceptability of mHealth apps—the use of the technology acceptance model to assess the acceptability of mHealth apps: systematic review. J. Med. Internet Res. 27:e66432. doi: 10.2196/66432, 40334265 PMC12096023

[ref2] Ahumada-Newhart V. Wood T. Satake N. Marcin J. P. (2024). Health perceptions and practices of a telewellness fitness program: exploratory case study. JMIR Format. Res. 8, e50710–e50710. doi: 10.2196/50710, 39622679 PMC11612521

[ref3] Al-Adwan A. S. Li N. Al-Adwan A. Abbasi G. A. Albelbisi N. A. Habibi A. (2023). Extending the technology acceptance model (TAM) to predict university students’ intentions to use metaverse-based learning platforms. Educ. Inf. Technol. 28, 15381–15413. doi: 10.1007/s10639-023-11816-3, 37361794 PMC10140721

[ref4] Bandura A. (1978). Self-efficacy: toward a unifying theory of behavioral change. Adv. Behav. Res. Ther. 1, 139–161. doi: 10.1016/0146-6402(78)90002-4 847061

[ref5] Brislin R. W. (1970). Back-translation for cross-cultural research. J. Cross-Cult. Psychol. 1, 185–216. doi: 10.1177/135910457000100301

[ref6] Cai J. Zhao Y. Sun J. (2022). Factors influencing fitness app users’ behavior in China. Int. J. Hum.-Comput. Interact. 38, 53–63. doi: 10.1080/10447318.2021.1921483

[ref7] Chen C.-C. Lin Y.-C. (2018). What drives live-stream usage intention? The perspectives of flow, entertainment, social interaction, and endorsement. Telemat. Inform. 35, 293–303. doi: 10.1016/j.tele.2017.12.003

[ref8] Chen X. Zhu Y. Xu X. (2024). Being there and being with them: the effects of visibility affordance of online short fitness video on users’ intention to cloud fitness. Front. Psychol. 15:1267502. doi: 10.3389/fpsyg.2024.1267502, 38362244 PMC10867133

[ref9] Davis F. D. Bagozzi R. P. Warshaw P. R. (1989). User acceptance of computer technology: a comparison of two theoretical models. Manag. Sci. 35, 982–1003. doi: 10.1287/mnsc.35.8.982, 19642375

[ref10] Delone W. H. McLean E. R. (2003). The DeLone and McLean model of information systems success: a ten-year update. J. Manag. Inf. Syst. 19, 9–30. doi: 10.1080/07421222.2003.11045748, 37339054

[ref11] Elsotouhy M. M. Ghonim M. A. Alasker T. H. Khashan M. A. (2024). Investigating health and fitness app users’ stickiness, WOM, and continuance intention using S-O-R model: the moderating role of health consciousness. Int. J. Hum.-Comput. Interact. 40, 1235–1250. doi: 10.1080/10447318.2022.2135813

[ref12] Fornell C. Larcker D. F. (1981). Evaluating structural equation models with unobservable variables and measurement error. J. Mark. Res. 18, 39–50. doi: 10.2307/3151312

[ref13] Frazier P. A. Tix A. P. Barron K. E. (2004). Testing moderator and mediator effects in counseling psychology research. J. Couns. Psychol. 51, 115–134. doi: 10.1037/0022-0167.51.1.115

[ref14] Guo J. Li L. (2022). Exploring the relationship between social commerce features and consumers’ repurchase intentions: the mediating role of perceived value. Front. Psychol. 12:775056. doi: 10.3389/fpsyg.2021.775056, 35401287 PMC8990309

[ref15] Henseler J. Ringle C. M. Sarstedt M. (2015). A new criterion for assessing discriminant validity in variance-based structural equation modeling. J. Acad. Mark. Sci. 43, 115–135. doi: 10.1007/s11747-014-0403-8

[ref16] Holt D. J. DeTore N. R. Aideyan B. Utter L. Vinke L. Johnson D. S. . (2024). Enhancing social functioning using multi-user, immersive virtual reality. Scientific Reports. 15. doi: 10.21203/rs.3.rs-4707220/v1 PMC11754853 39843493

[ref17] Hu L. Bentler P. M. (1999). Cutoff criteria for fit indexes in covariance structure analysis: conventional criteria versus new alternatives. Struct. Equ. Model. 6, 1–55. doi: 10.1080/10705519909540118

[ref18] Jacoby J. (2002). Stimulus-organism-response reconsidered: an evolutionary step in modeling (consumer) behavior. J. Consum. Psychol. 12, 51–57. doi: 10.1207/S15327663JCP1201_05

[ref19] Jensen H. R. (2001). Antecedents and consequences of consumer value assessments: implications for marketing strategy and future research. J. Retail. Consum. Serv. 8, 299–310. doi: 10.1016/S0969-6989(00)00036-9

[ref20] Kaveladze B. T. Morris R. R. Dimitrova-Gammeltoft R. V. Goldenberg A. Gross J. J. Antin J. . (2022). Social interactivity in live video experiences reduces loneliness. Front. Digital Health 4:859849. doi: 10.3389/fdgth.2022.859849, 35403096 PMC8989841

[ref21] Kline R. B. (2011). Principles and Practice of Structural Equation Modeling. New York: Guilford Publications.

[ref22] Kundu S. Datta S. K. (2015). Impact of trust on the relationship of e-service quality and customer satisfaction. EuroMed J. Bus. 10, 21–46. doi: 10.1108/emjb-10-2013-0053

[ref23] Lei S. Wu Q. Du J. (2025). How music tempo influences consumer preferences for advertising with different regulatory focuses: shopping in jumping tempo. J. Advert. Res. 65, 579–596. doi: 10.1080/00218499.2025.2464277

[ref8001] López-Carril S. Bae D. Ribeiro T. Alguacil M. (2025). Social media as a driver of physical activity: A snapshot from sport sciences students. Perform. Enhanc. Health 13:100331. doi: 10.1016/j.peh.2025.100331

[ref24] Mehrabian A. Russell J. A. (1974). An Approach to Environmental Psychology. Cambridge: MIT Press.

[ref25] Miao J. Ma L. (2022). Students’ online interaction, self-regulation, and learning engagement in higher education: the importance of social presence to online learning. Front. Psychol. 13:815220. doi: 10.3389/fpsyg.2022.815220, 36312116 PMC9613360

[ref26] Mrvelj Š. Matulin M. Martirosov S. (2020). Subjective evaluation of user quality of experience for omnidirectional video streaming. Promet – Traffic Transp. 32, 409–421. doi: 10.7307/ptt.v32i3.3444

[ref27] Rodrigues Sampaio A. Pimenta N. Machado M. Teques P. (2020). Development and validation of the fitness coaching behavior scale: factor structure, validity and reliability. Retos: Nuevas Perspectivas De Educación Física, Deporte Y Recreación 37, 683–693. doi: 10.47197/retos.v37i37.74344

[ref28] Su J. Liu H. Chen Y. Wang M. Zhang J. (2026). Enhancing product selection in live-streaming E-commerce: a multi-criteria decision-making framework. J. Retail. Consum. Serv. 88:104538. doi: 10.1016/j.jretconser.2025.104538

[ref29] Sung E. Mayer R. E. (2012). Five facets of social presence in online distance education. Comput. Hum. Behav. 28, 1738–1747. doi: 10.1016/j.chb.2012.04.014

[ref30] Sweeney J. C. Soutar G. N. (2001). Consumer perceived value: the development of a multiple item scale. J. Retail. 77, 203–220. doi: 10.1016/S0022-4359(01)00041-0

[ref31] Taherdoost H. (2018). A review of technology acceptance and adoption models and theories. Procedia Manuf. 22, 960–967. doi: 10.1016/j.promfg.2018.03.137

[ref32] Te A. Y. C. (2022). “The process of making the choice: three phases and the factors of influence,” in Choosing Chinese Universities: A negotiated choice for Hong Kong students (Chapter 8). (Abingdon, Oxon New York, NY: Routledge).

[ref33] Tian Y. Frank B. (2024). Optimizing live streaming features to enhance customer immersion and engagement: a comparative study of live streaming genres in China. J. Retail. Consum. Serv. 81:103974. doi: 10.1016/j.jretconser.2024.103974

[ref34] Ulaga W. Chacour S. (2001). Measuring customer-perceived value in business markets. Ind. Mark. Manag. 30, 525–540. doi: 10.1016/S0019-8501(99)00122-4

[ref35] Wang L. Ma Y. Yan Z. Zhang L. Hu Y. Zhao S. (2025). Giving or receiving: impact of online socializing in online fitness community on physical activity and emotional state. Comput. Hum. Behav. 169:108669. doi: 10.1016/j.chb.2025.108669

[ref36] Wang M. Wang Z. Deng R. (2025). How to enhance generation Z users’ satisfaction experience with online fitness: a case study of fitness live streaming platforms. Front. Comput. Sci. 6:1499672. doi: 10.3389/fcomp.2024.1499672

[ref37] Wongkitrungrueng A. Assarut N. (2020). The role of live streaming in building consumer trust and engagement with social commerce sellers. J. Bus. Res. 117, 543–556. doi: 10.1016/j.jbusres.2018.08.032

[ref38] Ye S. Lei S. I. Shen H. Xiao H. (2020). Social presence, telepresence and customers’ intention to purchase online peer-to-peer accommodation: a mediating model. J. Hosp. Tour. Manag. 42, 119–129. doi: 10.1016/j.jhtm.2019.11.008

[ref39] Yin M. Xiao R. (2025). VIBES: exploring viewer spatial interactions as direct input for livestreamed content. Proceedings of the 2025 ACM International Conference on Interactive Media Experiences. p. 200–219

[ref40] Yu J. Wang L. Zhang W. Zhou C. (2025). Winning markets via live-streams: competitive manufacturers’ channel strategies. J. Retail. Consum. Serv. 87:104391. doi: 10.1016/j.jretconser.2025.104391

[ref41] Zeithaml V. A. (1988). Consumer perceptions of price, quality, and value: a means-end model and synthesis of evidence. J. Mark. 52, 2–22. doi: 10.1177/002224298805200302

[ref42] Zhang T. Zhao J. Yu L. (2023). The effect of fitness apps usage intensity on exercise adherence among Chinese college students: testing a moderated mediation model. Psychol. Res. Behav. Manag. 16, 1485–1494. doi: 10.2147/PRBM.S408276, 37138699 PMC10150761

[ref43] Zhou W. Amirpour H. (2025). Perceptual visual quality assessment in multimedia communication. Proceedings of the 33rd ACM International Conference on Multimedia. p. 14340–14341

[ref44] Zhou J. Zhou J. Ding Y. Wang H. (2019). The magic of danmaku: a social interaction perspective of gift sending on live streaming platforms. Electron. Commer. Res. Appl. 34:100815. doi: 10.1016/j.elerap.2018.11.002

